# Cancer experience in metaphors: patients, carers, professionals, students – a scoping review

**DOI:** 10.1136/spcare-2024-004927

**Published:** 2024-05-13

**Authors:** Yufeng Liu, Elena Semino, Judith Rietjens, Sheila Payne, On behalf of 4D PICTURE Consortium, Amara Callistus Nwosu

**Affiliations:** 1 Department of Linguistics and English Language, Lancaster University, Lancaster, UK; 2 Department of Public Health, Erasmus MC, University Medical Centre Rotterdam, Rotterdam, the Netherlands; 3 Department of Design, Organisation and Strategy, Faculty of Industrial Design Engineering, Delft University of Technology, Delft, the Netherlands; 4 International Observatory on End of Life Care, Lancaster University, Lancaster, UK

**Keywords:** Cancer, Communication, Cultural issues, Family management

## Abstract

The use of metaphors to talk about cancer experiences has attracted much research and debate, especially in the case of military metaphors. However, questions remain about what metaphors are used by different populations for different aspects of the cancer experience. This scoping review aims to answer them.

We searched PubMed, PsycINFO, CINAHL, Scopus and Web of Science databases. Eligible sources include peer-reviewed scientific research published in English between 2013 and 2023, investigating patterns of metaphor use from adult populations (age ≥18) for cancer-related topics, such as cancer itself, the general experience of being ill, treatment, and people and relationships.

Out of 1929 articles identified, 30 met the criteria, spanning over different populations. While most papers focused on cancer in general, some focused on specific cancer types, such as breast cancer. Both spontaneous and elicited data were collected in ten languages: mostly English (N=12), Swedish (N=3) and Arabic (N=3). The identified metaphors were subsumed under various broad categories, including particularly violence and journey. Other categories include education and non-human animate entity for the cancer itself, confinement and deprivation and cleanliness for the general experience of being ill with cancer, Poison and gardening for cancer treatment, and distance for patients’ social relationships.

It was found that metaphors help to identify how patients describe experiences of vulnerability and empowerment. To provide patient-centred care, clinicians and researchers should avoid blanket conclusions about helpful or unhelpful metaphors, but consider the ways in which different metaphors are used by different populations in different contexts.

**WHAT IS ALREADY KNOWN ON THIS TOPIC**

- Metaphors are both linguistic and conceptual phenomena.
- Different metaphors frame the topic in different ways, highlighting some aspects and backgrounding others.
- Patients, carers and health professionals use metaphors to talk about the experience of cancer, and particularly violence or fight-related metaphors, which have been found to be potentially problematic.

**WHAT THIS STUDY ADDS**

- Previous studies treat cancer experience as a whole, but metaphors are used to capture different aspects of it, including the cancer itself, the general experience of being ill, treatment and people and relationships.
- Violence and journey metaphors are the most used by all populations, but they can capture different aspects of the cancer experience. Patients, carers and health professionals use violence metaphors in an attempt to get adequate care for patients.
- A variety of other metaphors are used by each population. Patients use education metaphors for the positive aspects of having cancer and distance metaphors for the consequence of cancer for relationships with family and friends.
- Metaphors in Arabic, Malay and Spanish tend to be related to religion.

**HOW THIS STUDY MIGHT AFFECT RESEARCH, PRACTICE OR POLICY**

- Metaphors can communicate different population groups’ perspectives on various aspects of the cancer experience.
- Future research can focus on the influence of demographic features (age, gender, ethnicity and religion) on the perception of cancer experiences; the potential impact of different cancer types on the metaphors employed by diverse populations and the collection of data in languages other than English.

## Introduction

 Recognised by the WHO as the second most prominent contributor to global mortality, accounting for one out of every six deaths in 2018,^1^ cancer is a subject of considerable scholarly interest across different academic disciplines. One non-clinical strand of research is concerned with the use of metaphors in communication about cancer experiences. This is because, in spite of long-standing debates about war-related metaphors, in particular,^2^ they are a central and almost unavoidable part of communication about cancer.^3^,^5^

There is no single definition of metaphor that is shared within the literature on metaphors for cancer. However, most studies are influenced by Lakoff and Johnson’s^6^ view of metaphor as a matter of thought as well as communication and language. They defined ‘conceptual metaphors’ as mappings between a ‘source’ conceptual domain (e.g., war) and a ‘target’ conceptual domain (e.g., illness). Conceptual metaphors are realised by and explain the existence of conventional metaphorical expressions, or linguistic metaphors, such as, for illness is a war, ‘fighting/battling/beating’ cancer, depression, dementia, etc. Crucially, the choice of source domain influences the way in which the target domain is understood, by ‘highlighting’ some aspects and ‘backgrounding’ others.^6^ The term ‘framing’ is used to capture this process, whereby the choice of metaphor facilitates particular types of reasoning, inferences, evaluations and emotional reactions with regard to the relevant target domain or topic.^7^

For example, it has been found that being exposed to the metaphor of cancer as a battle leads to the attribution of greater feelings of guilt to the sick person if they do not get better than being exposed to the metaphor of cancer as a journey.^8^ This is consistent with the framing of not recovering as ‘losing the battle’. It has also been noted, however, that the same metaphors may be empowering or disempowering for different people, depending on who uses them and how.^4^ For example, describing oneself as a ‘fighter’ can emphasise optimism and determination, and therefore, present the person as empowered in their approach to the illness.

Scholars in different disciplines, such as linguistics, social work, medicine and palliative care, have studied the metaphors employed by patients with cancer, carers, health professionals and other relevant groups to communicate about cancer experiences.^9^,^11^ Yet, the extent to which they employ similar or distinct research designs and the resulting metaphors they identify need to be explored. Furthermore, few studies^11^ have explored which specific metaphors are employed by which populations to describe which aspects of the cancer experience. To address these knowledge deficits, we conducted a scoping literature review aiming to answer two research questions: (1) What is the extent and nature of published scientific literature on metaphors describing cancer experiences? (2) Within this literature, what metaphors have been identified to portray different aspects of the cancer experience, and how do these metaphors vary among different population groups?

## Method

### Design

The review was conducted following the methodological framework outlined by Arksey and O’Malley for scoping the literature,^12^ supplemented by the updated scoping review methodological guidance provided by Peters *et al*.^13^ We opted for this review approach to obtain a comprehensive overview of the breadth and depth of literature on using metaphors to communicate about cancer experiences. In reporting our findings, we adhered to the Preferred Reporting Items for Systematic Reviews and Meta-Analyses Extension for Scoping Reviews.^14^ The scoing review protocol was uploaded as online supplemental file 1.

### Search strategy

We performed an extensive search across five different electronic databases: PubMed, PsycINFO, CINAHL, Scopus and Web of Science, in August 2023. The search process was iterative, with search terms and strategies adapting as we became more familiar with the literature. After numerous iterations, we established that employing the search query ‘metaphor* AND cancer*’ in titles, abstracts and keywords across the databases proved to be highly effective. This approach yielded comprehensive results, encompassing a wide array of cancer-related publications, including those pertaining to ‘chemotherapy’, ‘metastasis’, ‘leukaemia’, ‘lymphoma’, ‘malignancy’, ‘tumour’ and ‘oncology’. To enhance the methodology, we supplemented the results by exploring internet resources including Google Scholar and our university library One Search.

### Selecting articles for inclusion

After a comprehensive review of the publications retrieved, we opted to focus our attention on publications released in or after 2013. This decision was prompted by two considerations: first, this period observed a steady annual output of no less than 40 publications, signifying a notable upswing in interest in this subject compared with previous years, with the publication number mostly below 10, occasional peaking round 30; second, this time frame allows us to gain a decade-long perspective on the subject matter.

Out of all search outputs, only peer-reviewed scientific articles written in English were considered. However, there were also other inclusion and exclusion criteria applied to the analysis, as listed in table 1. For instance, only articles that drew from informal, unedited language data collected from participants aged 18 and older were included in the later, more fine-grained qualitative analysis. We excluded articles that focused on literary texts, such as fictions, dramas and poetry. We focused on verbal metaphors rather than visual metaphors in our attempt to explore the ways in which different populations discuss cancer experiences in words.

**Table 1:** Inclusion and exclusion criteria

Inclusion and exclusion criteria	
Inclusion criteria	Exclusion criteria
Studies summarise metaphors used to describe cancer-related topics, including cancer itself, patients, health professionals, carers, etc.	The author(s) propose the metaphor(s) without supporting data from the populations.
Participants are over 18 years old.	Studies are labelled as ‘articles’ in the databases, but they are, in fact, scientific review papers that do not contain informal, unedited data.
Studies draw from informal, unedited language data.	Studies examine cancer as the source domain, rather than the target domain.
The identified metaphors are verbal rather than visual.	Studies work on the methodological issue, specifically focused on demonstrating how to identify metaphors in cancer discourse.
The publication was written in English.	Empirical research primarily involves statistical analyses to assess the effectiveness or impact of specific metaphor usage, often comparing battle and journey metaphors.
The publication was released on or after the year 2013.	Studies use artwork, photographs or published poetry as their research materials
The publication is a peer-reviewed scientific article.	

Studies that treated cancer as the source domain, describing other subject matters as cancer-like, were excluded.^15^ Studies solely dedicated to methodological issues related to metaphor identification^16^ or controlled experiments designed to test the effectiveness of predetermined cancer metaphors^17^ were also excluded.

Following an initial round of screening where publications failing to meet the specified criteria for publication year, language and type were excluded, the remaining English-language peer-reviewed scientific articles published from 2013 to 2023 (inclusive) were transferred to the Rayyan platform^18^ for further analysis and management.

Two reviewers independently conducted screenings of all titles and abstracts of the publications imported into Rayyan, based on the inclusion and exclusion criteria listed in table 1. Publications were categorised as ‘included’, ‘excluded’ or ‘uncertain’ based on the scoping nature of this review. Subsequently, we collaborated to review and discuss the coding results. In instances where disagreements persisted after discussion, a third reviewer was invited to provide guidance. Throughout this process, the full text was consulted when deemed necessary.

### Data extraction and analysis

Once the included cases were definitively identified, the corresponding full texts were retrieved for further assessment. To facilitate this evaluation, a standardised data extraction form was used, encompassing various elements such as authors, publication year, journal discipline, type of cancer under investigation, the language of the data, study location, research objectives, the nature of the data, study population, a summary of metaphors in the study and the aspects of the cancer experience being examined. The data were extracted based on a close reading of the articles, supplemented by discussions with coauthors.

Inevitably, the authors of the reviewed studies used different approaches to classifying the linguistic metaphors semantically or thematically in their data. For example, the word ‘fight’ was labelled as a Battle metaphor in one study^19^ and as a war/fight metaphor in another study.^9^ Some studies did not subsume the metaphors under the relevant source domain but grouped them according to the broad themes they were used to express, such as ‘isolation, marginalisation and self-isolation’ and ‘managing identity’ in Montali *et al*^20^ and Appleton and Flynn.^21^ In online supplemental tables for the results section, we report the original labels or theme in a column titled ‘Metaphor label in original study’. We also provide our own labels in a column titled ‘Metaphor label in current study’, to bring together semantically related instances of metaphor that received different labels in the original studies. For example, we use the label violence metaphors for the metaphors that the authors of the studies subsumed under war/fight, Battle and so on. In most cases, our labels are more generic versions of the original labels. In a few cases, we have reused the original labels, as they were formulated at the appropriate level of abstraction (e.g., when the original label was violence metaphors). In a small number of cases, the original labels did not seem to account adequately for the associated linguistic example (e.g., the linguistic metaphor ‘A worse pain will be waiting for me that will take me away’ was described as ‘the metaphor of living in dark future with pain’).^22^ In such cases, our labels reflect our own approach to classification based on the literal meanings of the metaphorically used words (e.g., ‘take me away’ was subsumed under confinement and deprivation). Overall, this approach allows us to capture broader patterns across studies employing different types of classifications for linguistic metaphors.

When considering the linguistic examples provided in each study, we combined two existing metaphor identification procedures^23 24^ to check that they qualified as metaphors or similes according to the best practices in metaphor studies. For example, the expression ‘battle’ in ‘battle against (cancer)’^19^ is identified as metaphorical because its meaning in context (‘trying to recover from cancer’) contrasts with a more basic meaning of the word (‘a clash between armies in a war’), but the former can be understood via comparison with the latter (ie, trying to recover from a serious illness can be understood in terms of a battle against an enemy in war).

If the authors do not provide quotations from the data to support their classification of the metaphors elicited from the participants in their studies, we will exclude them from the results section, such as the studies by Lemmo *et al*,^25^ Fergus *et al*^26^ and Kırca and Kaş.^27^

All these methodological considerations result from an observation that the selected papers are multidisciplinary in nature, and therefore, the ways they define and label metaphors are different. For instance, there are cases where the authors did not explicitly mention how they defined and labelled metaphors.^9 21 22 25 28^ In addition, some studies, though claiming to employ the metaphor identification procedure^19 29^ or systematic metaphor analysis,^20^ did not follow them rigorously.

Finally, to answer research question 2, we classified the metaphors according to the aspect of the cancer experience that they are used to capture (e.g., the cancer itself, treatment). This classification is used to structure the overview of metaphors in the results section. When reporting the results in onlinesupplemental tables 2^5^, we italicise the metaphorical words that capture the relevant aspect of the cancer experience and anonymise the names in each example. However, the same example may contain metaphors illustrating different aspects of the cancer experience. In such cases, we may include the same example in different tables. In some cases, we provide multiple examples from the same study in order to do justice to the variety of linguistic expressions that may realise the same broad type of metaphor. Nevertheless, this review is not a systematic reanalysis of all the data in the reviewed studies.

## Results

**Figure 1:**
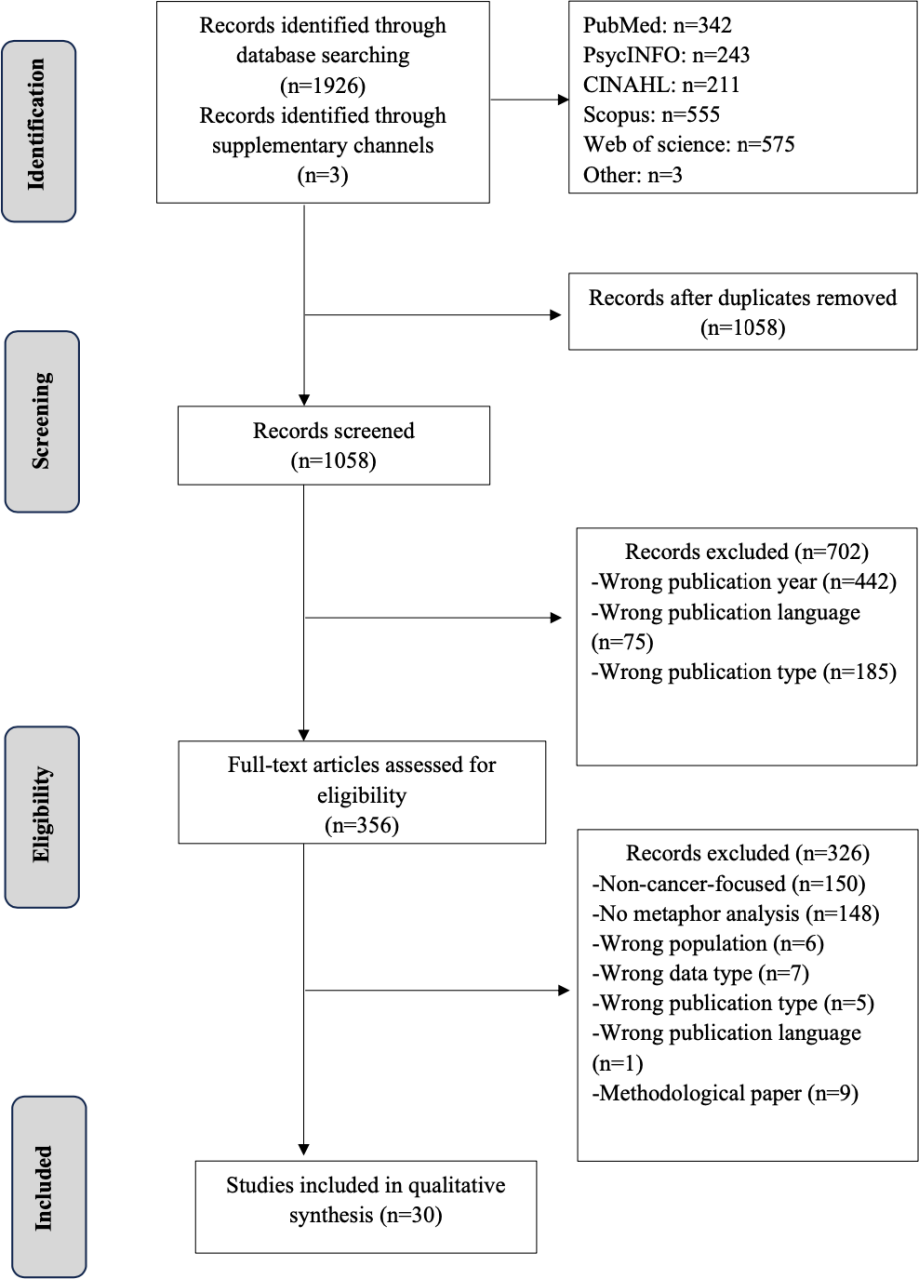
PRISMA flow diagram of literature search and selection. PRISMA, Preferred Reporting Items for Systematic Reviews and Meta-Analyses.

The search strategy produced a total of 1929 results. After a thorough critical appraisal, 30 papers were identified as pertinent (see figure 1). Among them, 16 papers were published in medical journals, 7 in linguistic journals, 1 in a marketing journal, 2 in psychology journals, 2 in social work journals and 2 in multidisciplinary journals. These papers primarily focus on cancer in general (n=15), with fewer addressing the breast (n=5), gynaecological (n=2), non‐Hodgkin’s lymphoma (n=1), ovarian (n=1), oesophageal (n=1) cancer or a combination of cancers (n=5). The data consist of both spontaneous sources, such as blogs, online forums, recorded conversations between nurses and patients with cancer, as well as elicited sources, including semistructured interviews and focus group discussions. In the elicited data, two cases prompted participants to produce metaphors using a predetermined question ‘cancer is…, because…’.^26 27^ Data were mostly in English (n=12), followed by Swedish (n=3), Arabic (n=3), Turkish (n=3), Spanish (n=1), Danish (n=1), Portuguese (n=1), Malay (n=1) and a mix of languages (n=4). One paper did not provide information on the language of the collected data.^22^ 22 papers gathered data from patients with cancer, 4 from a mixed population, 2 from nursing students and 2 from individuals without health issues.

A summary of characteristics of the included 30 papers, including author details, publication year, journal discipline, cancer type, language of data, research location, objectives, research data, study population and a summary of metaphors in the original study, is provided in online supplemental table 1. However, as stated in the ‘Data extraction and analysis’ section, we will classify the metaphors into broader categories and distinguish the aspects of cancer experience they refer to in subsequent sections.

### Metaphors for the cancer itself

In this section, we consider metaphors for the cancer itself. These are realised linguistically by metaphorically used nouns that, in context, refer to cancer, such as the noun ‘teacher’ in the example ‘I continue the fight with this disease that I consider today my great *teacher’*.^30^ Online supplemental table 2 presents the corresponding results.

These metaphors can be subsumed under 12 broad categories: violence, journey, education, religious test, gift, nature, human in general, invasion of personal space, non-human animate entity, burden, cognitive stimuli and other. Overall, a broad distinction can be made between metaphors that present the cancer as an animate being and metaphors that present it as an inanimate entity or phenomenon. The metaphors we have subsumed under violence, human in general, and, in one case, education, present the cancer as a human being, for example, ‘enemy’,^31^ ‘fellow’^31^ and ‘teacher’.^30^ In contrast, the metaphors under non-human animate entity present the cancer as an animal (‘octopus’),^32^ a fantastic creature (‘monster’)^19 30 33^ or a supernatural entity (e.g., ‘demon’).^29 34^

The metaphors in both violence and non-human animate entity groups tend to position the patient in a vulnerable and disempowered position in relation to the illness, as the illness is capable of scaring, hurting or killing them. This also applies, but to a lesser extent, to the metaphors under invasion of personal space, describing the cancer as an ‘unwanted tenant in my body’^35^ and as a ‘stranger’ who ‘comes back’ and ‘sits down by my side’.^19^ These metaphors involve the violation of personal space and potentially some sense of underlying threat, but without an element of physical aggression. In contrast, in the description of the cancer as a ‘teacher’^30^ under education, the cancer is still presented as being in a more powerful position than the patient, but the attitude that is attributed to it is benevolent.

The rest of the metaphors in online supplemental table 2 present the cancer as different kinds of inanimate entities or phenomena and also differ from one another in terms of their implications for the relationship between the person and the illness. When the cancer is described as a ‘test’, in the context of education^30 32^ or religion,^36^ the patient is in a disempowered position where they are under pressure to respond or behave appropriately. Lack of control on the patient’s part is also involved in some of the descriptions categorised under nature, that is, as ‘smoke’^37^ and a ‘tsunami’.^21^ The latter has similar implications of metaphorical physical damage as some of the metaphors discussed above under violence and non-human animate entity. In both cases, however, the natural phenomenon subsides over time (cf. ‘clearing waters’ and ‘later disperses’).

The metaphors classified under journey include a conventional allusion to death (‘the last journey of life’),^38^ and the description of the cancer as ‘a very dark tunnel’ that the person has ‘come out of’.^31^ The dark tunnel combines two elements—darkness and containment—that make metaphorical travel frightening and distressing. However, in context, the person is presented as being able to continue on the implied metaphorical journey beyond the difficulties caused by the cancer.

The metaphors we labelled under education, religious test, gift, human in general, invasion of personal space, burden and cognitive stimuli were exclusively used by patients. Three groups—education, religious test and gift—suggesting a relatively benevolent view of cancer, were identified in Spanish (*maestra* ‘teacher’),^30^ Turkish (‘test’),^32^ Arabic (*alla:h qad ḥalla bi:* ‘Allah’s decree’)^36^ and Malay (*pemberian* ‘gift’).^39^ Religious test and gift metaphors in Arabic and Malay mirror certain religious beliefs, as the ‘test’ or ‘gift’ is from Allah.

People with family members or friends affected by cancer tend to conceptualise cancer more conventionally as ‘an enemy in crossfire with the patient’s body’^38^ or ‘the last journey of life’.^38^ Nursing students compare cancer to a natural phenomenon (‘smoke’).^37^ Among them, those related to violence and journey were in Arabic, while those under non-human animate entity and other were in English. The ‘smoke’^37^ metaphor was identified in Turkish. The ‘monster’,^19 33^ ‘burden’^40^ and ‘stranger’^19^ metaphors under non-human animate entity, burden and invasion of personal space were identified in Swedish.

### Metaphors for generally being ill with cancer

In this section, we consider metaphors for the general experience of being ill with cancer (see online supplemental table 3). These metaphors fall under 12 broad categories: violence, journey, confinement and deprivation, water and danger, cleanliness, sports, machinery-driven movement, transformation, education, job, burden and other.

Violence and journey metaphors are the most frequently reported types of metaphors in the reviewed studies. Violence metaphors represent the relationship between the patient and the disease as antagonistic. When the patient is presented as ‘fighting’, these metaphors capture the effort and determination to get better (e.g., ‘confront’,^9 36^ ‘defeat’^19^ and ‘vanquish’^36 37^ the cancer). In such cases, the patient is placed in an agentive and potentially empowered position. In contrast, when the disease is presented as ‘attacking’, violence metaphors capture the negative physical and/or emotional consequences of being ill (e.g., ‘I’m afraid of the *violence* of this new *attack’*).^9^ In such cases, the patient is placed in a passive and disempowered position.

Journey metaphors mostly represent the experience of being ill as a lengthy and difficult process that requires perseverance (e.g., ‘We read, asked a lot of questions, and took it one *step* at a time’).^30 31^ With the exception of one case where the cancer is presented as travelling inside the body (‘*gone through* the lymph nodes’),^35^ within journey metaphors the patient is positioned as the traveller. There is variation, however, in terms of (a) whether the patient is travelling alone (in most cases) or alongside others (e.g., ‘although the *road* is tough, if you fall, we will help you get up!!!’)^30^; (b) whether they have control of the journey (e.g., ‘Thank God, I was able to *keep going’*^31^ vs ‘I want to *climb off* but there is *no stop button’*)^40^ and (c) whether the destination is recovery (e.g., ‘The *path* to recovery’)^36^ or death (e.g., ‘I felt terrified that the time of *departure* is approaching and I don’t know how much time is left for me’).^36^ The extent to which the patient is presented as active and empowered depends on the amount of control they have over the journey, both in terms of its progress and direction and in terms of their attitude towards it (e.g., ‘My *journey* may not be smooth but it certainly makes me look up and take notice of the scenery!’).^4^

For both violence and journey metaphors, instances of resistance have been identified, namely, case where patients explicitly reject a particular metaphor (‘not a courageous *fight’*, ‘it’s *not a journey’*).^9 21 28^

Sports metaphors share with violence metaphors the representation of the relationship with cancer as competitive (e.g., ‘overcoming cancer is very similar to *running a race* …’)^31^ while they share with Journey metaphors the representation of being ill as requiring perseverance (e.g., ‘It’s a *marathon’*).^32^ In all reported cases, the patient is placed in the relatively empowered position of being at least equal to the cancer as a sporting opponent.

Both transformation and education metaphors capture what some patients perceive as positive changes in their lives as a result of having cancer (e.g., ‘a rebirth’^29^ and ‘The disease *taught* me to value life’).^31^ In contrast, most of the remaining groups of metaphors in online supplemental table 3 place the patient in a disempowered position due to lack of control and associated negative emotions.

Confinement and deprivation metaphors capture the inability to experience life as the person did before the illness by presenting the cancer as a malevolent agent who deprives the person of things (e.g., ‘This illness has *taken everything away* from me’)^41^ or freedom (e.g., ‘It is as if someone had *put a free bird in a cage’*).^40^ Water and danger metaphors express lack of control and fear via scenarios in which the patient is in danger of drowning because of falling into dangerous waters (e.g., ‘As if I’ve *fallen into a slime lake’*)^32^ or being hit by waves (‘After diagnosis, my life *as a large wave rolling in’*).^42^ The metaphors we have subsumed under machinery-driven movement capture lack of control via scenarios in which the person cannot get off from a machine or vehicle that moves independently of their will (e.g., ‘I think once *you’re on (the treadmill*), you’re on it’).^43^ Metaphors involving lifts and fairground rides additionally capture extreme and uncontrollable changes in emotional states via rapid vertical movement, where ‘up’ is positive and ‘down’ is negative (e.g., ‘My feelings were *up and down like a lift*.’).^29^ Burden metaphors capture the difficulties and negative emotions associated with being ill via scenarios in which a heavy object makes movement difficult and pushes the person in a downward direction (e.g., ‘To have a relapse during ongoing treatment is *heavy’*).^40^

Finally, cleanliness metaphors use the opposition between clean and dirty to capture two different aspects of the experience of illness: the contrast between having or not having cancerous cells in one’s body (‘They gave me great and incredible news … I am *clean*!’)^31^ and the contrast between one’s values and priorities before and after the illness (‘I had to do a big *clean up* in my values and re-prioritise them’).^9^ The latter use of cleanliness metaphors places the person in an empowered position in relation to how they live their own lives, similarly to some of the metaphors discussed above.

Violence and journey metaphors were shared by both patients and other populations, including people with family members or friends affected by cancer and nursing students. They were found to be used in multiple languages: English, Arabic, Turkish, Swedish, French, Italian and Spanish.

Nine broad categories of metaphors were exclusively employed by patients: confinement and deprivation, water and danger, cleanliness, sports, machinery-driven movement, transformation, education, job and burden. Water and danger metaphors were from languages other than English, including Arabic, Turkish, Portuguese and Danish. Similarly, the machinery-driven movement metaphor was identified in Danish, Swedish and Arabic. Sports metaphors were identified in English, Spanish and Turkish. Confinement and deprivation metaphors were identified in Swedish; education and cleanliness in Spanish and transformation in Arabic

### Metaphors for treatment

In this section, we consider metaphors for treatment (see online supplemental table 4). Metaphors were found to be used to describe cancer treatment in general, specific types of treatment (chemotherapy, radiotherapy and surgery), decision-making in treatment, treatment consequences and the side effects of treatment.

Most of the metaphors for treatment in general, chemotherapy and radiotherapy capture the negative physical and emotional consequences of treatment. This applies to violence metaphors where the patient is at the receiving end of metaphorical aggression (e.g., ‘they *hit* me with radiation’)^34^ and to poison, wave and fire metaphors, which all involve metaphorical scenarios of physical harm (e.g., ‘Chemotherapy is *poison’*,^28^ ‘before the big *wave* hits you’^34^ and ‘my body was *set on fire’*).^36^ Here, the patient is in a similarly disempowered position as for some of the metaphors describing the experience of illness. In the same way, journey metaphors are used to present treatment generally and chemotherapy specifically as a lengthy and difficult process that requires perseverance (e.g., ‘a long arduous *journey’*).^35^

Other metaphors for treatment capture its benefits. This applies to violence metaphors that allude to the possibility of cure in terms of scenarios where the cancer is at the receiving end of aggression (e.g., ‘the treatment will serve to *defeat* this disease’),^30^ and to a gardening metaphor for the spiritual benefits of experiencing surgery (‘surgery was my heavenly father’s way of pruning me so I could blossom and grow in my spiritual life’).^30^ A fixing metaphor implies the possibility of restoring the patient’s body to health in the same relatively straightforward that applies to repairing a machine.^35^

Both metaphors for treatment decision-making express a preference for being guided by the expertise of healthcare professionals, whether via a vote scenario that alludes to the brexit referendum result in the UK (‘my vote’s 48 percent but yours is 52’) or via a Journey scenario where the healthcare professional ‘walks’ the patient to a decision.^10^

All but one metaphor in online supplemental table 4 were employed by patients with cancer. Journey metaphors were identified in English, Swedish and Spanish. The metaphors in the violence, poison, wave and fire groups were identified across a range of languages, including English, French, Arabic, Swedish and Spanish. The fixing metaphor was identified in English. Both gardening and gift metaphors were used in Spanish.

### Metaphors for people and relationships

In this section, we consider metaphors describing the people involved in the cancer experience (patients, family members and healthcare professionals) and their mutual relationships (see online supplemental table 5).

Violence metaphors account for approximately half of the metaphors in online supplemental table 5. When used by patients to describe themselves or one another, they involve expressions that present the person as empowered, that is, as strong, determined and likely to recover from cancer (e.g., ‘warrior’, ‘fighter’, ‘survivor’).^7 30 34 36 44^ In contrast, when violence metaphors are applied by patients to their relationship with healthcare professionals, they highlight the difficulties and effort involved in receiving the care they need (e.g., ‘twin *attack’*^4^ and ‘another thing to *beat* my surgeon *up* about’).^7^ The health professionals’ counterparts of these metaphors describe physicians as ‘generals’, their role as ‘protection’ and patients as ‘troops killed in battle’,^34^ combining benevolence with the positioning of patients as disempowered. The difficulties involved in dealing with health systems are also described via violence metaphors by health professionals and family carers, with members of both groups needing to ‘fight’ for adequate levels of care.^34^

The remaining metaphors in online supplemental table 5 were exclusively used by patients with cancer. De-humanising metaphors are used to express negative feelings or self-perceptions as a result of being ill. This applies to object metaphors (‘Honestly, I am *as tough as old boots’*),^45^ and zombie metaphors (‘I feel like a *zombie’*).^46^ One particular individual conveys the contrast between the anger and fatigue they feel and the impression of calm they give to others via a Volcano metaphor (‘everything was fine outside while inside I was a *volcano* in other words’) and an intertextual reference to Jekyll and Hyde.^20^ Finally, several different metaphors are used to convey the ways in which the cancer has affected patients’ relationships with friends and family members. Different sports metaphors capture collaboration (‘a team’) versus insensitive behaviour (‘She would just *bowl in* and she would be busy doing things’).^20^ Metaphors to do with physical distance similarly capture both the appreciation of intimacy and understanding (e.g., ‘the people *close* to me who gave me a lot of help’) and the loss of intimacy and contact (e.g., ‘I *withdrew* from a lot of people’ and ‘those who took […] a *lateral position* in my life’).^20^

Violence metaphors were identified in English, Spanish, Arabic and Italian. The metaphors presenting the patient as a non-human entity were identified in English, Italian and Danish while distance metaphors were identified in Italian.

## Discussion

### Summary of main findings

Our review has clarified the extent and nature of previous literature on the use of metaphors to describe cancer experiences. Specifically, we found the reviewed studies predominantly centred around cancer in general, with half of them (n=15) not reporting the cancer types under investigation. The remainder tends to focus on female-related cancer types, such as breast, ovarian, cervical, and gynaecological cancer in a broader sense (n=12). While around half of the studies collected data in English (n=12), other languages were also studied, including Swedish, Arabic, Turkish, Spanish, Danish, Portuguese, Malay, French and Italian. Over two-thirds of the studies collected data from patients with cancer, with smaller numbers of studies focusing on health professionals, carers, nurses and nursing students. The most frequently collected spontaneous data included blogs, online forums and recorded conversations between nurses and patients with cancer, whereas elicited data mostly consisted of semistructured interviews and focus group discussions.

Due to the multidisciplinary nature of the literature, there were different approaches to identification and to the classification of metaphors extracted from the data. Additionally, there was usually no attempt to distinguish between metaphors for different aspects of the cancer experience. We have pointed out distinctions between metaphors for the cancer itself, the general experience of being ill with cancer, treatment, and people and relationships.

The metaphors for cancer itself tend to present the cancer as either an animate being or an inanimate entity or phenomenon. In the former, the metaphors under the violence, non-human animate entity, invasion of personal space and education groups mostly place the cancer as a separate entity that is in a more powerful position than the patient, but the attitude attributed to the cancer can be malevolent or benevolent. In the latter case, the metaphors under religious test and nature mostly express the patients’ disempowerment and lack of control.

The metaphors for generally being ill with cancer mostly fall under two broad categories: violence and journey. Violence metaphors represent the relationship between the patient and the disease as antagonistic, with the patient placed either in an empowered or disempowered position. Journey metaphors tend to highlight the lengthy and difficult process with the patient positioned as the traveller. There were also cases of explicit resistance against both violence and journey metaphors. Metaphors classified under confinement and deprivation, water and danger, machinery-driven movement highlight the patient’s lack of control and fear, whereas metaphors under transformation and education emphasise positive changes in the patient’s lives.

The metaphors for cancer treatment mostly capture the adverse physical and emotional effects of undergoing treatment, as with violence, poison, wave and fire metaphors. Fixing and gardening metaphors capture the physical and spiritual benefits of treatment. Both vote and journey metaphors for treatment decision-making express a preference for being guided by the expertise of healthcare professionals.

The metaphors for people and relationships are mostly violence metaphors. Patients present themselves as active but not always empowered while health professionals describe their role as ‘generals’ who attempt to ‘protect’ the patients. All groups use violence metaphors to emphasise difficulties in dealing with health systems. Other metaphors tend to de-humanise the patients, such as object and zombie metaphors, or highlight the patient’s (lack of) intimacy and contact with others, such as distance metaphors.

Most studies focus on metaphors used by patients with cancer, resulting in a lack of consideration for other crucial stakeholders, including health professionals, carers and others. Notable exceptions are the studies by Demmen *et al*^34^ and Semino *et al*,^4^ where health professionals and carers are found to employ violence metaphors for their relationships with the healthcare system and the higher authorities within the system. People with family members or friends affected by cancer and nursing students were mostly found to use relatively conventional violence and journey metaphors to refer to the different aspects of the cancer experience. However, they also employed novel metaphors, such as referring to the cancer as ‘smoke’ that ‘spreads’.

Most studies focused on the use of metaphors in English. However, among metaphors for cancer itself, violence and journey metaphors were identified in Arabic. Among metaphors for the general experience of being ill with cancer, education and cleanliness were found in Spanish, confinement and deprivation in Swedish, machinery-driven movement in Danish, Swedish and Arabic, water and danger in Arabic, Turkish, Portuguese and Danish. Gardening and gift metaphors for cancer treatment were identified in Spanish, whereas distance metaphors for the patient’s social relationships were identified in Italian.

Nevertheless, we lack a relatively balanced view of how different populations in different countries with different demographic characteristics use and understand these metaphors. We also did not investigate how these metaphors affect the patients, carers, clinicians and others in various cancer scenarios, which needs a response-elicitation approach.

### Limitations of this review

While this scoping review provides valuable insights into the breadth and nature of the literature on using metaphors to describe cancer experiences over the past decade, it did not involve a systematic evaluation of each study’s quality or an assessment of how effectively the different metaphors communicate cancer experiences. The specified time frame, language criterion, publication type and databases pose a potential risk of overlooking some relevant studies. However, we ensured relatively reliable results in selecting articles for inclusion and data extraction and analysis as authors of this scoping review have linguistic and medical backgrounds, and worked independently and collaboratively to ensure the accuracy of data searching, extraction and analysis.

### Implications for future research

While the reviewed studies touch on metaphors from populations beyond patients with cancer, it is patients with cancer who receive a disproportionately high level of attention (n=22). Exploring how other key stakeholders, namely health professionals, carers and families, express their perceptions of cancer, life experiences and relationships—both within and across groups—will be essential for a comprehensive understanding of cancer communication.

The data analysed are predominantly in English, prompting the question of whether populations speaking different languages may employ different metaphors to communicate about cancer. It is well known that there are cross-linguistic and cross-cultural differences as well as similarities in conventional metaphors for a wide range of experiences and phenomena.^47^

Few demographic details were provided in the reviewed studies. Future research may consider investigating how individuals with different demographic attributes, including age, gender, ethnicity, religion, occupation and marital status, employ metaphors to describe their cancer experiences.

The cancer types under study are relatively limited, with a primary focus on cancer in a general sense and female-related cancer types. However, understanding the potential distinctions in using metaphors to describe experiences with various cancer types, and different stages of cancer, could add additional insights.

We strived to distinguish metaphors for the cancer itself, the general experience of being ill with cancer, treatment and people and relationships, but our categorisation is not exhaustive. Further studies are needed to collect metaphors describing cancer experiences beyond the scope of this review.

## Conclusions

In this scoping review, the identified metaphors are used by different populations (patients with cancer, health professionals, carers, etc) and they describe various aspects of the cancer experience (the cancer itself, the general experience of being ill with cancer, treatment and people and relationships), their predominant focus remains on understanding how patients with cancer perceive cancer and their general experience of being ill with cancer. The identified metaphors can be subsumed under various broad categories, with the violence and journey groups being dominant across the four aspects of the cancer experience. Other categories include education, religious test and non-human animate entity for the cancer itself, confinement and deprivation, water and danger and cleanliness for the general experience of being ill with cancer, poison, fire and gardening for cancer treatment, and the distance group for the patients’ social relationships. As cancer emerges as an increasingly urgent topic for people across the globe and given the influential role metaphors play in shaping and reflecting our views and emotions about cancer, there is a compelling need for future research in this domain. Future research may consider focusing on populations other than patients with cancer and, crucially, on languages other than English, given the global prevalence of cancer and the potential variations in understanding shaped by the socio-political and religious beliefs in different country contexts.

## supplementary material

10.1136/spcare-2024-004927 online supplemental file 1

10.1136/spcare-2024-004927 online supplemental file 2

10.1136/spcare-2024-004927 online supplemental file 3

10.1136/spcare-2024-004927 online supplemental file 4

10.1136/spcare-2024-004927 online supplemental file 5

10.1136/spcare-2024-004927 online supplemental file 6
