# Supplementary material for: Cancer experience in metaphors: patients, carers, professionals, students – a scoping review
Source: BMJ Support Palliat Care. 2024 May 13;14(e3):e004927. doi: 10.1136/spcare-2024-004927 (PMC11671970; doi:10.1136/spcare-2024-004927)
Supplement: online supplemental file 1 [file spcare-14-e3-s001.pdf]

Describing cancer experiences using metaphors: A scoping review

A SCOPING REVIEW PROTOCOL

Prepared for a peer-review process

Submitted April 9th, 2024

REVIEW TITLE AND TIMESCALE

Review Title: Describing cancer experiences using metaphors: A scoping review

Anticipated/actual start date: 25/08/2023

Anticipated/actual completion date: 12/03/2024

Stage of review at time of this submission

**Review Stage**	**Started**	**Completed**
Preliminary searches	☐	✓
Piloting of the study selection process	☐	✓
Formal screening of search results against eligibility criteria	☐	✓
Data extraction	☐	✓
Risk of bias (quality assessment)	☐	N/A
Data analysis	☐	✓

REVIEW TEAM DETAILS

Named contact: Dr. Yufeng Liu

Named contact address: CASS centre, County South, Lancaster University, England, LA1 4YL

Review team members and their organization affiliations:

**Title**	**Affiliation**
Dr. Yufeng Liu	Department of Linguistics and English Language, Lancaster University, Lancaster, UK
Prof. Elena Semino	Department of Linguistics and English Language, Lancaster University, Lancaster, UK
Prof. Judith Rietjens	Faculty of Industrial Design Engineering, TU Delft, Netherlands
Prof. Sheila Payne	Division of Health Research, Lancaster University, Lancaster, UK

Funding sources/sponsors: The research leading to these results has received funding from EU research and innovation programme HORIZON Europe 2021 under grant agreement 101057332 and by the Innovate UK Horizon Europe Guarantee Programme, UKRI Reference Number 10041120.

Conflicts of interest: Authors have no known conflicts of interest to declare.

REVIEW METHODS:

Review Questions:

This scoping review aims to identify metaphors used to describe different aspects of the cancer experience in previous studies. Specifically, it answers the following research questions:

1) What is the extent and nature of published scientific literature on metaphors describing cancer experiences?

2) Within this literature, what metaphors have been identified to portray different aspects of the cancer experience, and how do these metaphors vary among different population groups?

Literature search:

Comprehensive literature searches of electronic databases were primarily completed by one author, with consultations from two co-authors to maximize sensitivity; a broad search strategy included databases: PubMed, PsycINFO, CINAHL, Scopus and Web of Science, in accordance with the PRISMA statement. Search terms were piloted several times for different databases. Since the major focus is on metaphors describing cancer-related topics, the pilot searches consider multiple keywords that are related to cancer: “cancer”, “carcinoma”, “malignancy/malignant”, “neoplasm*”, “tumor*/tumour”, “Oncology”, “Oncologist” “Carcinogen”, “Sarcoma”, “metastasis/metastases”, “Melanoma”, “Leukemia”, “Lymphoma”, “Myeloma”.

In each database, we conducted searches using the following search strings in sequence:

“metaphor* AND cancer*”, “metaphor* AND carcinoma*”, “metaphor* AND malignan*”, “metaphor* AND neoplasm*”, “metaphor* AND tumor*”, “metaphor* AND tumour*”, “metaphor* AND oncolog*”, “metaphor* AND Carcinogen*”, “metaphor* AND Sarcoma*”, “metaphor* AND metastas*”, “metaphor* AND Melanoma*”, “metaphor* AND Leukemia*”, “metaphor* AND Lymphoma*”, “metaphor* AND Myeloma*”.

It turns out that the search string “metaphor* AND cancer*” sufficed the requirements in every database, as the results obtained from this string encompassed those derived from the additional search strings. Consequently, we chose to employ the search string “metaphor* AND cancer*” to compile data from the databases.

Searches were conducted in titles, abstracts, or keywords. All language entries were included initially. The searched results were saved. Two authors then independently analysed the titles, abstracts, and keywords (sometimes the full texts) for studies’ eligibility. A third opinion was consulted for ambiguous cases.

Condition of domain being studies:

Metaphors describing cancer experiences.

Participants/population:

The participants/population under investigation are adults, including cancer patients, health professionals, nurses, carers, and nursing students.

Types of study to be included initially:

Peer-reviewed empirical papers.

Context:

Papers published between 2013 and 2023 (included).

Data extraction (selection and coding):

Information was extracted by two independent researchers. When necessary, for cases of ambiguity or discrepancy, a third researcher reviewed the article to discern if the article meets the inclusion criteria.

Risk of bias (quality) assessment:

Since this is a scoping review, a quality appraisal will not be conducted.
