# Supplementary material for: Cancer experience in metaphors: patients, carers, professionals, students – a scoping review
Source: BMJ Support Palliat Care. 2024 May 13;14(e3):e004927. doi: 10.1136/spcare-2024-004927 (PMC11671970; doi:10.1136/spcare-2024-004927)
Supplement: online supplemental file 2 [file spcare-14-e3-s002.pdf]

Supplementary Table 1a. The nature and extent of published scientific literature on metaphor and cancer—Population: Cancer patients

Study	Journal discipline	Cancer Type	Language of data	Study location	Study aims	Methodology	Study population	Metaphor results
Abaalalaa & Ibrahim 2022[36]	Linguistics	Breast cancer	Arabic	Arabic countries	to explore the use of metaphors in the narratives of breast cancer patients in online magazine websites in the Arabic language	Cancer patients' stories from magazines, newspapers and cancer institutions (13,705 words)	19 females with breast cancer No ages given	War; Journey; Trial by Ordeal/Test of faith; Support; Transformation of character
Almegewly & Alsoraihi 2022[29]	Psychology	Breast cancer	Arabic	Saudi Arabia	to investigate how Saudi Arabian women express their experiences with breast cancer using metaphors	Semi-structured interviews	18 breast cancer patients; aged 30-53; in a premenopausal status; finished treatment 6–47 months before interviews took place; with no current history of mental illness or advance cancer stage (metastasis)	Dark hidden force; Battling imminent death; Dreaming and awakening calls; Inner and outer transformation
Appleton & Flynn 2014[21]	Medical	Cancer in general	English	UK	to investigate how the language and metaphors of cancer influence personal and social adjustment after completion of a course of treatment	Transcribed focus group recordings (elicited participants' stories and focused discussion on key words and common phrases in the cancer lexicon)	A focus group of 18 people whose course of active treatment for cancer had ended; 11 females, 7 males; aged 45-85	Journey; Survivor
Aydın *et al.* 2022[32]	Medical	Gynaecological cancer	Turkish	Turkey	to explore the experiences of gynaecological cancer patients during the COVID-19 pandemic from their own perspectives	Semi-structured interviews	17 females with gynaecological cancer and receiving chemotherapy; aged 35-63	War; Marathon; Octopus; Test; Devastation; Slime; Sea voyage; Slide

Supplementary Table 1a *Continued*

Study	Journal discipline	Cancer Type	Language of data	Study location	Study aims	Methodology	Study population	Metaphor results
Bodd *et al.* 2023[28]	Medical	Breast, lung, and colorectal cancer	English	USA	(1) frequency and use of metaphors to describe cancer diagnosis, treatment, or survivorship; (2) function and impact of the war metaphor on the patient experience of cancer.	Semi-structured interviews	15 participants with either breast, lung, or colorectal cancer; having an active treatment plan (radiation, hormonal therapy, immunotherapy, or chemotherapy); mean age 59.6, SD (12.6)	Journey; Nature; War; Uncertainty
Chircop & Scerri 2018[41]	Medical	non-Hodgkin's lymphoma	English	Unknown	to explore the use of metaphors by non-Hodgkin's lymphoma (NHL) patients undergoing chemotherapy in a haematology ward setting	Semi-structured interviews (17,079 words)	6 adult patients diagnosed with NHL and undergoing chemotherapy; 4 males and 2 females; mean age 63	War; Prison; Journey
Fergus *et al.* 2017[26]	Multidisciplinary	Breast cancer	English	Canada	to facilitate shared meaning construction in young couples affected by breast cancer	An online artistic expression exercise: Step 1: "To us, cancer is like a because ..."; Step 2: to develop a creative portrayal of their metaphor on a virtual white board using icons, pictures, photos, colours and/or letters available via the Couplelinks platform	13 females diagnosed with breast cancer and their male partners	Detour; Sea

Supplementary Table 1a *Continued*

Study	Journal discipline	Cancer Type	Language of data	Study location	Study aims	Methodology	Study population	Metaphor results
Guité-Verret & Vachon 2021[9]	Medical	Breast cancer	French or English	Canada, United States, France	to better understand the experience of four women with incurabale metastatic breast cancer from the metaphors they used in personal cancer blogs	Cancer patients' blogs	4 women who had been diagnosed with stage 1 to 3 breast cancer in the past; aged 35-53	The fight; The unveiling
Gustafsson & Hommerberg 2018[33]	Linguistics	Cancer in general	Swedish	Sweden	to shed light on this battle script by examining the discursive dynamics of metaphor use in a large corpus of Swedish blogs written by terminally ill patients.	Cancer patients' blogs (approximately 2,600,000 words)	27 patients with a terminal cancer diagnosis; 21 females, 6 males; aged 20-70; mean age 40	Kämpa ('fight'/'struggle'); Ge Upp [give up]
Gustafsson *et al.* 2020[19]	Medical	Advanced breast, colon, gynaecological and other cancer	Swedish	Sweden	to explore how bloggers with advanced cancer use metaphors as ways of making sense of their experiences.	Cancer patients' blogs	27 patients with a terminal cancer diagnosis; 21 females (median age 36) and 6 males (median age 44)	Battle; Journey; Imprisonment
Hommerberg *et al.* 2020[40]	Medical	Advanced breast, colon and gynaecological cancer	Swedish	Sweden	to investigate the use of metaphors in blogs written in Swedish by people living with advanced cancer and explore possible patterns associated with individuals, age and gender.	Cancer patients' blogs (2,602,479 words)	27 patients with a terminal cancer diagnosis; 21 females (median age 36) and 6 males (median age 44)	Journey; Battle; Imprisonment; Burden

Supplementary Table 1a *Continued*

Study	Journal discipline	Cancer Type	Language of data	Study location	Study aims	Methodology	Study population	Metaphor results
Laranjeira *et al.* 2015[42]	Social work	Gynecological Cancer	Portuguese	Portugal	to provide a deeper understanding of the temporal existential experiences of women who are living with gynecological cancer	Interviews	10 Portugese gynecological cancer survivors; aged 33-70, mean age 55; 4 with cervical cancer, 4 with endometrial cancer, 2 with ovarian/fallopian tube cancer	Large wave rolling in (Tsunami); Feeling off time; Being shifted off a perceived normal life course trajectory
Laursen *et al.* 2019[46]	Medical	Oesophageal cancer	Danish	Denmark	to illuminate the ways in which incurable oesophageal cancer disrupts the patients' lives and how the patients experience and adapt to life with the disease	Semi-structured interviews	17 patients receiving palliative care for oesophageal cancer; 7 females and 10 males; aged 54-74; 1–23 months after the cancer diagnosis	Zombie; Feeling at sea
Magaña 2020[30]	Medical/ Communication	Breast cancer	Spanish and English	USA/Spain	to analyse the Latina and Spanish women's use of metaphor to express patients' and survivors' attitudes, fears, and concerns about cancer as well as the advice they offer to others	Cancer stories (totaling 29,967 words, with about 10,000 words each in Spanish by U.S. Latinas, English by U.S. Latinas, and Spanish by Spanish females)	51 females with breast cancer; aged 30-78	229 conceptual metaphors: Violence (56%); Journey (34%); Other (10%)

Supplementary Table 1a *Continued*

Study	Journal discipline	Cancer Type	Language of data	Study location	Study aims	Methodology	Study population	Metaphor results
Magaña & Matlock 2018[31]	Linguistics	Cancer in general	Spanish	Spain, Argentina, Mexico or other Spanish-speaking countries	to expand prior work on cancer communication by examining a language other than English, by focusing on how cancer victims communicate among themselves, and by examining how cancer narratives discuss concerns about personal experiences with this disease	60 online cancer narratives (37,180 words)	60 cancer survivors and patients; 12 males, 48 females; all adults (no ages given)	Violence (75%); Journey (19%); Other (6%)
Malmström *et al.* 2018[43]	Medical	Colorectal and lung cancer	Danish, English, Swedish	Denmark, England and Sweden	to explore how the emphasis on early diagnosis and timely treatment is reflected in patient's accounts of care, from the first suspicion of colorectal or lung cancer to their treatment in Denmark, England and Sweden	Semi-structured narrative interviews	155 lung or colorectal cancer patients within six months of diagnosis; aged 31-90; females 36-50% of the total	Kinetic metaphors (such as rollercoaster, treadmill)
Mijomanović 2015[35]	Linguistics	Cancer in general	English	Unknown	to examine metaphors used by cancer patients that either represent their views of the cancer itself or the emotions evoked by this experience	Cancer patients' personal stories	English speaking cancer patients; no gender split; no ages given	Cancer is War; Patient is a Vehicle; Cancer is a Journey; Cancer is a Tunnel/Detour/Limited Journey; Cancer is an (Unwanted) Inhabitant; Cancer is Force; Body is Container/Machine
Mohd Jamil *et al.* 2019[39]	Linguistics	Cancer in general	Malay	Malaysia	to examine the use of the OBJECT metaphor in Malay women's narratives on cancer	Semi-structured interviews (28,052 words)	11 females with different types and stages of cancer; aged 20-56	Cancer is a Gift; Cancer is an Unwanted Object; Cancer is a Heavy Object

Supplementary Table 1a *Continued*

Study	Journal discipline	Cancer Type	Language of data	Study location	Study aims	Methodology	Study population	Metaphor results
Montali *et al.* 2023[20]	Medical	Ovarian cancer	English and Italian	Australia and Italy	to deepen knowledge of the impact of ovarian cancer on patients' social relationships by analysing the metaphors that patients use to narrate their illness.	Semi-structured interviews	14 Australian and 24 Italian females diagnosed at different stages of ovarian cancer; aged 32-80	The authors did not name any metaphor, but identified four themes that were metaphorically described: Lack of comprehension and communication; Isolation, marginalisation and self-isolation; Discrepancy between the private and public self; Social relationships as empowerment resources
Raiisi & Riyassi 2022[22]	Medical	Cancer in general	Unknown	Iran	to investigate the lived experience of pain metaphors in cancer patients	Semi-structured interviews	20 patients in stages 2 and 3 of the cancer; 11 females, 9 males; mean age: 43.37	The metaphor of lived war: fighting together; The metaphor of living in dark future with pain; The metaphor of lived with overload pain
Semino *et al.* 2018[7]	Linguistics	Cancer in general	English	UK	to bring together cognitive and discourse-based approaches in an integrated multi-level framework for metaphor analysis, and demonstrate its value to both theory and practice by applying it to a corpus-based study of violence-related metaphors for cancer	A 15,000-word sample from a UK-based online forum for patients with cancer (500,134 words)	56 patients with cancer; no gender split; no ages given	Violence, Journey, Other
Sinding 2014[10]	Social work	Cancer in general	English	Canada	to examine metaphors that emerged from one patient's reflections on what was missing, and what she wished were different, in her interactions with cancer care professionals.	One cancer patient Sheila's narrative	One cancer patient Sheila; no age given	Voting; Walking me there

Supplementary Table 1b The nature and extent of published scientific literature on metaphor and cancer—Population: Other

Study	Journal discipline	Cancer Type	Language of data	Study location	Study aims	Methodology	Study population	Metaphor results
Albarghouthi & Klempe 2019[38]	Psychology	Cancer in general	Arabic	The West Bank area of the occupied Palestinian territories	to understand the sociocultural complexity and social representations (SR) of cancer in the occupied Palestinian territory: (1) SRs of cancer and cancer treatment, (2) paradoxical SRs of cancer patients, and (3) religious discourses and SRs of cancer	Semi-structured interviews	16 participants; 10 males and 6 females; aged 26-83; 7 had close family members with cancer, 9 had indirect experiences through friends or neighbors; 14 Muslims, 2 Christians	War-related/Militaristic metaphors; Journey; Personification
Demmen *et al.* 2015[34]	Linguistics	Cancer in general	English	UK	1. to demonstrate an approach to the study of metaphor patterns in large data- sets that combines manual qualitative analysis with quantitative semi-auto- mated corpus methods;<br>2. to provide a systematic analysis of variation in the frequencies, forms and functions of the Violence metaphors used by the three main stakeholder groups in cancer care and end-of-life care: patients, family carers and health professionals.	The MELC (Metaphor in End-of-Life Care) corpus: approximately 300,000 words collected from semi-structured interviews and approximately 1.2 million words sampled from online forum contributions, totalling approximately 1.5 million words.	i. 16 senior health professionals working in hospices or hospital-based palliative care;<br>ii. 29 patients with a diagnosis of terminal cancer;<br>iii. 17 unpaid family carers looking after family members who have a terminal diagnosis.<br>iv. online forum users	Violence
Kırca & Kaş 2022[27]	Medical	Cancer in general	Turkish	Turkey	to reveal the perceptions of the concept of cancer among second-year nursing students through metaphors.	Interview forms with a fixed formula: Cancer is…, because…	65 second-year nursing students in the Nursing Department of the Faculty of Health Sciences of a Turkish university	44 different metaphors, the most common ones: Fight, Test, Love

Supplementary Table 1b *Continued*

Study	Journal discipline	Cancer Type	Language of data	Study location	Study aims	Methodology	Study population	Metaphor results
Lanceley & Clark 2013[45]	Medical	Cancer in general	English	UK	to identify the opportunities patients take in conversation to express their feelings, how they articulate them to the nurses, and the nature of their disclosures, elucidating the links between personal meaning and emotional expression	60 conversations between nurses and cancer patients	21 nurses, 60 cancer patients; 46 females and 14 males; aged 20–81	Euphemistic death metaphor; Cancer is a surprise; The body is tough as old boots; Cancer is hiccups in my breast; Cancer is an ugly creature; Game
Lemmo *et al.* 2022[25]	Medical	Breast and cervical cancer	English	Italy	to understand what the relationship with preventive practices in oncology means for and how this relationship is revealed by their metaphors.	Invented stories by answering a narrative prompt	58 young women under the age of 45 in university groups	Disease as extraction of a number or a name; Family ghost; The laceration of the body attacked and filled with poison; The uncontrollable invader; Breast and uterus were twins, and when one got angry, the other tried to reassure it by passing its energy and strength to it; The metaphor of the waiting room emerges as a merciless purgatory; To be lifted as if being filled with helium; To stay on the road with an extra file in order and one less pebble in the shoe; Being a woman who stops at the zip of her jeans
Semino *et al.* 2017[4]	Medical	Cancer in general	English	UK	to compare the frequencies with which patients with cancer and health professionals use Violence and Journey metaphors when writing online; and to investigate the use of these metaphors by patients with cancer, in view of critiques of war-related metaphors for cancer and the adoption of the notion of the 'cancer journey' in UK policy documents.	A UK-based online forum for patients with cancer (500,134 words);<br>A UK-based website for health professionals (253,168 words)	56 patients with cancer; 307 health professionals; no gender split; no ages given	Disempowering and empowering violence metaphors; Disempowering and empowering journey metaphors

Supplementary Table 1b *Continued*

Study	Journal discipline	Cancer Type	Language of data	Study location	Study aims	Methodology	Study population	Metaphor results
Torres & DeBerry-Spence 2019[44]	Marketing	Cancer in general	English	USA	to explore the relationship between consumer valorization and traumatic extraordinary experiences, hereafter referred to as traumatic experiences	458 archived blog posts; field notes from participation in and observations of cancer-related events (128 text pages); 16 informal interviews with non-bloggers; 24 institutional websites	213 cancer patient bloggers, aged 17-72; Participants in eight cancer-related events and activities (12 to 500-plus participants each); 16 non-blogger interviewees, aged 20-65 (5 patients, 4 clinicians, 4 representatives from nonprofit cancer support organizations, and 3 caregivers)	War; Journey
Yesilbalkan *et al.* 2021[37]	Medical	Cancer in general	Turkish	Turkey	to define cancer from the perspective of Turkish nursing students	Questionnaire (1. demographic information; 2. description of the concept of cancer; 3. a written elicitation meeting to find the justifications for cancer metaphors)	166 fourth-year nursing students at a nursing faculty in Turkey	92 valid metaphors, the most common ones: Death; War; Virus; Malady; and Spider
