# Supplementary material for: Cancer experience in metaphors: patients, carers, professionals, students – a scoping review
Source: BMJ Support Palliat Care. 2024 May 13;14(e3):e004927. doi: 10.1136/spcare-2024-004927 (PMC11671970; doi:10.1136/spcare-2024-004927)
Supplement: online supplemental file 3 [file spcare-14-e3-s003.pdf]

Supplementary Table 2 Metaphors for cancer itself in selected papers

Metaphor label in current study	Metaphor label in original study	Example	Studies
Violence	Violence	But the emotional side of cancer and of BC [breast cancer] in particular is the real *killer*—it strangles and shocks your soul.	Magaña 2020[30]<br>Semino *et al.* 2017[4]<br>Semino *et al.* 2018[7]
	Violence	Un primer aprendizaje para nosotros fue que el enemigo a vencer era la enfermedad. [A first lesson for us was that the disease was the *enemy* to beat.]	Magaña & Matlock 2018[31]
	War	In addition, cancer was named as *enemy*, with the body caught in crossfire.	Aydın *et al.* 2022[32]<br>Albarghouthi & Klempe 2019[38]<br>Bodd *et al.* 2023[28]
Journey	Journey	Ahora le debo muchísimo a ella porque gracias a ella salí de ese túnel tan negro. [Now I owe a lot to her because thanks to her I came out of that very *dark tunnel*.]	Magaña & Matlock 2018[31]
	Journey	It (cancer) means the last *journey* in life.	Albarghouthi & Klempe 2019[38]
Education	Teacher	[I continue the fight with this disease that I consider today my great *teacher*. It is what forced me to sit in the front row to watch a movie about myself. Something my soul needed so badly.]	Magaña 2020[30]
	Test/Challenge	[Through this grueling *test*, I learned to respect and forgive others.]	Magaña 2020[30]
	Test	I think it's like two big *tests*, it really affected me. But whatever God says happens.	Aydın *et al.* 2022[32]

Supplementary Table 2 *Continued*

Metaphor label in current study	Metaphor label in original study	Example	Studies
Religious Test	Trial by Ordeal (Test of Faith)	I touched the mass and examined the tumour. I immediately realised that I was afflicted by *Allah's decree*.	Abaalalaa & Ibrahim 2022[36]
Gift	Gift	Saya banyak belajar untuk redha dengan pemberian Al-lahni. [I have learnt a lot to embrace this *gift* from Allah.]	Mohd Jamil *et al.* 2019[39]
Nature	―― (Theme: Managing emotions)	To me I just see in the face of what is a *tsunami* and then it got to March, April, I see it's clearing waters ahead, that's the way I try to look at it.	Appleton & Flynn 2014[21]
	Nature	This is not my whole life, it's a part of my life, *a season of my life* that will pass regardless of if it goes terribly bad and I pass away—God forbid—it is still not the entirety of my life. I had 38 years up to this point of my life.	Bodd *et al.* 2023[28]
	Smoke	It's *like smoke*, it *spreads* quickly but later disperses…	Yesilbalkan *et al.* 2021[37]
Human in General	Personification	La biopsia fue concluyente: Linfoma de Hodgkin, ¡con mucho gusto! Jamás había escuchado hablar de tal 'señor' ... conocer el nombre y apellido contra quien peleaba, me generó una sensación de tranquilidad. [The biopsy was conclusive: Hodgkin Lymphoma, nice to meet you! I had never heard about this *fellow* ... knowing the first and last name of whom I was fighting against, gave me a peace of mind.]	Magaña & Matlock 2018[31]
Burden	Burden	I've been carrying this *burden* for four years now. Always this backpack... (W58).	Hommerberg *et al.* 2020[40]

Supplementary Table 2 *Continued*

Metaphor label in current study	Metaphor label in original study	Example	Studies
Non-Human Animate Entity	Octopus	Like an *octopus*. It comes towards me with all its arms.	Aydın *et al.* 2022[32]
	Dragon	Un saludo y muchos ánimos porque podemos ganar a ese dragon. [Greetings and lots of encouragement because we can defeat that *dragon*.]	Magaña & Matlock 2018[31]
	Dark hidden force (demon/evil)	Cancer looked like a *demon* was trying to kill me.	Almegewly & Alsoraihi 2022[29]<br>Demmen *et al.* 2015[34]
	Dark hidden force (demon/evil)	I'm afraid of the *devil's eye*.	Almegewly & Alsoraihi 2022[29]
	Battling imminent death	The *phantom* of cancer is still present.	Almegewly & Alsoraihi 2022[29]
	Monster	(T)hey gave me the results...and there is still no trace/sign of the *monster*.	Magaña 2020[30]<br>Gustafsson & Hommerberg 2018[33]<br>Gustafsson *et al.* 2020[19]

Supplementary Table 2 *Continued*

Metaphor label in current study	Metaphor label in original study	Example	Studies
Invasion of personal space	Personification	The illness [...] is present in my entire existence. Only gone brief moments. Then it comes back like a *stranger* and sits down by my side.	Gustafsson *et al.* 2020[19]
	Unwanted inhabitant	Cancer is an *unwanted tenant* in my body, but I am doing all I can to stay positive and protect myself with healthy living, exercise and the right diet, which will, hopefully, help to ensure that I will able to throw it out again, if necessary.	Mijomanović 2015[35]
	Object	Saya tak nak benda (kanser) tu dalam badan saya. [I don't want that *thing* (cancer) in my body.]	Mohd Jamil *et al.* 2019[39]
	An unwanted object	Saya tak nak benda tu (cancer) ada dalam badan saya, saya nak buang. [I don't want that *thing* (cancer) to be in my body, I want to throw it (remove) away.]	Mohd Jamil *et al.* 2019[39]
Cognitive Stimuli	Uncertainty (e.g., mystery, puzzle)	There's so much information out there, but still there's no cure that is applicable to all the people... It works for some people, it doesn't work for others... It's like a *puzzle*.	Bodd *et al.* 2023[28]
	Surprise	So you see all this has been rather a *surprise* but I'd never I've never been ill so it's very difficult.	Lanceley & Clark 2013[45]
	Dreaming and awakening calls	This disease was like a *wakeup call*.	Almegewly & Alsoraihi 2022[29]
Other	Hiccups in the breast	*hiccups* in my breast…	Lanceley & Clark 2013[45]
