# Supplementary material for: Cancer experience in metaphors: patients, carers, professionals, students – a scoping review
Source: BMJ Support Palliat Care. 2024 May 13;14(e3):e004927. doi: 10.1136/spcare-2024-004927 (PMC11671970; doi:10.1136/spcare-2024-004927)
Supplement: online supplemental file 4 [file spcare-14-e3-s004.pdf]

Supplementary Table 3 Metaphors for being ill with cancer generally in selected papers

Metaphor label in current study	Metaphor label in original study	Example	Studies
Violence	Battling imminent death	The woman is on a *fight* with a cancer to live, to survive.	Almegewly & Alsoraihi 2022[29]
	Unarmed war	*Unarmed war*. Two great *enemies* and there is nothing you can do ...	Aydın *et al.* 2022[32]
	War/fight	I've been *fighting* all my life. I feel like one more *fight* won't make a difference...	Abaalalaa & Ibrahim 2022[36]<br>Albarghouthi & Klempe 2019[38]<br>Appleton & Flynn 2014[21]<br>Aydın *et al.* 2022[32]<br>Bodd *et al.* 2023[28]<br>Chircop & Scerri 2018[41]<br>Gustafsson & Hommerberg 2018[33]<br>Guité-Verret & Vachon 2021[9]<br>Mijomanović 2015[35]<br>Torres & DeBerry-Spence 2019[44]<br>Yesilbalkan *et al.* 2021[37]
	War/fight	So I suppose *survival* to me feels a privilege. I'm sort of realistic. I try very hard to live in the minute.	Appleton & Flynn 2014[21]<br>Guité-Verret & Vachon 2021[9]<br>Mijomanović 2015[35]<br>Torres & DeBerry-Spence 2019[44]
	War/fight	True examples of a group of heroes who have *defeated* the malignant, and managed to *vanquish* cancer with will power and challenge.	Abaalalaa & Ibrahim 2022[36]<br>Yesilbalkan *et al.* 2021[37]
	War/fight	I said to myself: I will not *surrender* to this disease because I love life.	Abaalalaa & Ibrahim 2022[36]<br>Albarghouthi & Klempe 2019[38]

Supplementary Table 3 *Continued*

Metaphor label in current study	Metaphor label in original study	Example	Studies
Violence	War/fight	I won the first *battle* in my disease	Abaalalaa & Ibrahim 2022[36]<br>Aydın *et al.* 2022[32]<br>Guité-Verret & Vachon 2021[9]<br>Torres & DeBerry-Spence 2019[44]
	War/fight	It was a cruel *struggle*.	Abaalalaa & Ibrahim 2022[36]<br>Gustafsson & Hommerberg 2018[33]<br>Yesilbalkan *et al.* 2021[37]
	War/fight	Dear breast cancer patient: be strong, *confront* cancer and don't let it *beat* you.	Abaalalaa & Ibrahim 2022[36]<br>Guité-Verret & Vachon 2021[9]
	War/fight	...cancer as[is] an "*invasion*" whereby cancer cells "*colonize*" the body.	Albarghouthi & Klempe 2019[38]
	War/fight	If you don't completely *kill* everything, then it actually gets stronger.	Bodd *et al.* 2023[28]
	War/fight	The *bomb exploded* ... I felt a total wreck.	Chircop & Scerri 2018[41]
	War/fight	I'm afraid of the *violence* of this new *attack*. I'm afraid our *chemical weapons* are not strong enough to *retaliate*.	Guité-Verret & Vachon 2021[9]
	War/fight	The support group members helped each other to live with and *conquer* their disease by sharing their experiences and feelings.	Mijomanović 2015[35]

Supplementary Table 3 *Continued*

Metaphor label in current study	Metaphor label in original study	Example	Studies
Violence	War (about pain)	The pain was *shooting* in my head.	Raiisi & Riyassi 2022[22]
	Battle	It takes so much energy to be in pain, energy that I need to be able to keep *fighting* and be skilful in my *battle* against [Fairy tale enemy].	Gustafsson *et al.* 2020[19]<br>Hommerberg *et al.* 2020[40]
	Battle	The cancer that I shall *defeat*!	Gustafsson *et al.* 2020[19]
	Violence	Two months later they detected a papillary thyroid tumor, which made it clear that something was not right with me, but I decided to *fight* tooth and nail.	Demmen *et al.* 2015[34]<br>Magaña 2020[30]<br>Magaña & Matlock 2018[31]<br>Semino *et al.* 2017[4]<br>Semino *et al.* 2018[7]
	Violence	Cancer cannot make me *surrender*.	Magaña 2020[30]
	Violence	Physical change was not easy to assimilate, but we must appreciate that the loss of hair and other effects are minor harms if we take into account that the treatment will serve to *defeat* this disease.	Magaña 2020[30]<br>Magaña & Matlock 2018[31]<br>Semino *et al.* 2017[4]
	Violence	I knew I was facing the *battle* of my life, and I prayed to win this *battle*.	Demmen *et al.* 2015[34]<br>Magaña 2020[30]<br>Magaña & Matlock 2018[31]<br>Semino *et al.* 2017[4]<br>Semino *et al.* 2018[7]
	Violence	After the third cancer diagnosis, I was very sad because I realized that cancer was slowly *invading* my body.	Magaña 2020[30]<br>Semino *et al.* 2017[4]

Supplementary Table 3 *Continued*

Metaphor label in current study	Metaphor label in original study	Example	Studies
Violence	Violence	I don't intend to give up; I don't intend to *give in*.	Demmen *et al.* 2015[34]
	Violence	I don't want it to *beat* me, I want to *beat* it.	Demmen *et al.* 2015[34]<br>Magaña & Matlock 2018[31]
	——<br>(Theme: Isolation, marginalisation, and self-isolation)	And so you ask the doctor and he tells you that you have to cancel that network of relationships, because this is *a battle that you do alone*.	Montali *et al.* 2023[20]
	——<br>(Theme: Managing emotions)	The *fight*, I mean conjures up that you are boxing it. you know, sort of *beating* it back you know and if it's sort of an anatomical thing.	Appleton & Flynn 2014[21]
	Devastation	*Fighting* against the two is, of course, like destruction, greater destruction. I cannot see my friends, and I have to face everything myself.	Aydın *et al.* 2022[32]
	Militaristic metaphors	Some patients are strong to *fight* it (cancer), but mostly they just *surrender*.	Albarghouthi & Klempe 2019[38]
	(Not) war/fight	So far I have always thought that treating cancer is *not* a courageous *fight*, but an imposed necessity.	Bodd *et al.* 2023[28]<br>Guité-Verret & Vachon 2021[9]

Supplementary Table 3 *Continued*

Metaphor label in current study	Metaphor label in original study	Example	Studies
Journey	Journey	My *journey* may not be smooth but it certainly makes me look up and take notice of the scenery!	Abaalalaa & Ibrahim 2022[36]<br>Appleton & Flynn 2014[21]<br>Bodd *et al.* 2023[28]<br>Gustafsson *et al.* 2020[19]<br>Hommerberg *et al.* 2020[40]<br>Laranjeira *et al.* 2015[42]<br>Magaña & Matlock 2018[31]<br>Mijomanović 2015[35]<br>Semino *et al.* 2017[4]
	Journey	I want to *climb off* but there is *no stop button*. No way of *making the carriage stop* so I just have to *ride* along.	Hommerberg *et al.* 2020[40]
	Journey	I felt terrified that the time of *departure* is approaching and I don't know how much time is left for me.	Abaalalaa & Ibrahim 2022[36]
	Journey	The *path* to recovery necessarily has moments of desperation and weakness and probably frustration.	Abaalalaa & Ibrahim 2022[36]<br>Magaña & Matlock 2018[31]
	Journey	Por muy complicada que esté la cosa puede brillar una luz al final del túnel. [However complicated the thing may be, a light may shine *at the end of the tunnel*.]	Magaña 2020[30]
	Journey	My husband and I became very educated on breast cancer and its treatment. We read, asked a lot of questions, and took it one *step* at a time.	Magaña 2020[30]<br>Magaña & Matlock 2018[31]
	Journey	...we're *headed out* together ...	Torres & DeBerry-Spence 2019[44]

Supplementary Table 3 *Continued*

Metaphor label in current study	Metaphor label in original study	Example	Studies
Journey	Journey	Piensa también que estás en manos de profesio- nales y que, aunque el camino es duro, si te caes, ¡te ayudaremos a levantarte!!! [Also, don't forget that you are in the hands of professionals and that although the *road* is tough, if you fall, we will help you get up!!!]	Magaña 2020[30]
	Journey	Gracias a Dios que se ha podido salir adelante. [Thank God, I was able to *keep going*.]	Magaña 2020[30]<br>Magaña & Matlock 2018[31]
	Journey	One patient comments that "*rocks* in our *paths* are easier to handle when we're all in it together", and that "the best people to help you are the ones who've been there before or are *heading* there with you".	Semino *et al.* 2017[4]
	Journey	Y me tocó aparcar mi vida ... [And I had to *park* my life ...]	Magaña & Matlock 2018[31]
	Journey	Ánimo a todas las personas que estéis atravesando estos duros momentos. [Courage to all of those people who are *crossing* those difficult times.]	Magaña & Matlock 2018[31]
	Journey	Aunque haya piedras en el camino, lo importante es saber que se pueden esquivar. [Although there are *bumps on the way*, it is important to know that you can dodge them.]	Magaña & Matlock 2018[31]
	Journey	The same grade three, *gone through* the lymph nodes and who – five years *down the track* – was still alive. Once I did speak to that lady, my life changed. I thought, "I can do it. I can be *there* too," and I am.	Mijomanović 2015[35]

Supplementary Table 3 *Continued*

Metaphor label in current study	Metaphor label in original study	Example	Studies
Journey	Sea voyage<br>Death is down	An endless *sea journey* ... there is also the *wind*, and it *shakes you from every direction*. However, if my condition *turns "South"* I will return to [PLACE], per my discussions with both Dr. [NAME] and Dr. [NAME].	Aydın *et al.* 2022[32]<br>Mijomanović 2015[35]
	(Not) journey	I absolutely hate that word, it's *not a journey*, it's an illness, a journey is somewhere you go nice on holiday	Appleton & Flynn 2014[21]
Confinement and Deprivation	Prison	This illness has *taken everything away* from me. Has taken everything away from me. Everything, everything ... I cannot go out, I cannot cook, I cannot eat, I cannot do housework.	Chircop & Scerri 2018[41]
	The metaphor of lived with overload pain	This pain had *taken* my breath *away*.	Raiisi & Riyassi 2022[22]
	The metaphor of living in dark future with pain	A worse pain will be waiting for me that will *take* me *away*.	Raiisi & Riyassi 2022[22]
	Imprisonment	It is as if someone had *put a free bird in a cage*. Taken away its freedom and left if without alternative to affect its destiny.	Hommerberg *et al.* 2020[40]
	Imprisonment	[Family name] is *holding me in a firm grip*. He refuses to *let go*, the stubborn bastard.	Gustafsson *et al.* 2020[19]
	Imprisonment	It's so bloody trying, both mentally and physically to feel as I do at the moment. I just want to *crawl out of my skin* and *escape* far, far away.	Gustafsson *et al.* 2020[19]
	Imprisonment	My conscious me, the me that loves and lives, the me that consists of all those experiences that define me is *captured* in a body that I hate. This is not how my body should be, I should not be *limited* like this. That this is the price I have to pay to stay alive is just to accept unconditionally.	Gustafsson *et al.* 2020[19]

Supplementary Table 3 *Continued*

Metaphor label in current study	Metaphor label in original study	Example	Studies
Confinement and Deprivation	Force	So this big *wall went up* and I thought, 'Oh, I wasn't expecting this', and all I talked about was how I could get out of the car park and where I could get some change – I didn't want to know about the cancer.	Mijomanović 2015[35]
	—— (Theme: managing identity)	It *puts* us *in* a cancer *box.*	Appleton & Flynn 2014[21]
Water and Danger	Battling imminent death	I felt I was *drowning*.	Almegewly & Alsoraihi 2022[29]
	Slime	As if I've *fallen into a slime lake*, the higher I want to go, the more they pull me in ...	Aydın *et al.* 2022[32]
	Wave allegory	After diagnosis, my life *as a large wave rolling in*, after that other [waves] problems appeared . . .	Laranjeira *et al.* 2015[42]
	Feeling at sea	When I started getting worse, I thought, Who should I contact? If you rang up, anyone could pick up the phone. That's what it felt like to me, anyway. It felt like there was no-one specific to refer to, someone who knew my case. I felt abandoned – *at sea*. There was no-one to talk to.	Laursen *et al.* 2019[46]
Cleanliness	A renewed look	I did a tune-up and I had to do a big *clean up* in my values and re-prioritize them. I think we should all take a moment to *clean up* our lives, we shouldn't wait for an ordeal to force us to do it.	Guité-Verret & Vachon 2021[9]
	The state of having cancer as being dirty	Me dieron la buena e increíble noticia ... ¡de que estoy limpia! [They gave me great and incredible news ... I am *clean*!]	Magaña & Matlock 2018[31]

Supplementary Table 3 *Continued*

Metaphor label in current study	Metaphor label in original study	Example	Studies
Sports	Game	but er it's just the build-up it's the build-up if you were a *sportsman* and you or *a rugby team* or whatever it's the build-up to erhm er (nurse sneezes) oops you've still got your cold haven't you oh no.	Lanceley & Clark 2013[45]
	Running a race	Y es que superar un cáncer tiene similitudes con correr una carrera ... [And overcoming cancer is very similar to *running a race* ...]	Magaña & Matlock 2018[31]
	Marathon	There are two people I fight, there are two people I compete with. And if one side wins it means bad. It's a *marathon*.	Aydın *et al.* 2022[32]
	Journey	To constantly create goals and subgoals between X-ray, assessment and results. Thinking in terms of *laps* makes it possible to regain the power over life. Feels grand.	Gustafsson *et al.* 2020[19]
Machinery-Driven Movement	Inner and outer transformation	My feelings were *up and down like a lift*.	Almegewly & Alsoraihi 2022[29]
	Kinetic metaphors	I think once *you're on [the treadmill]*, you're on it, and that you're being dealt with really. And I suppose I maybe am one of the lucky ones, that I was dealt with quickly.	Almegewly & Alsoraihi 2022[29]
	Oscillation	Cancer is a *ride* that takes me through many *twists* and *turns*, *ups* and *downs*, *highs* and *lows*. Part of me wants to always be positive, to look at the good and downplay the bad. At the same time, it wouldn't be honest to do that.	Guité-Verret & Vachon 2021[9]
	Journey	So while it's fantastic to know I made the 5 years seemingly successfully, it sucks equally as bad to know that I'm back in line waiting for the next *roller coaster ride* to begin.	Torres & DeBerry-Spence 2019[44]
	Kinetic metaphors	But it was a *rollercoaster* from, I think you got cancer to the op, it was, bum, bum, bum, bum, bum and that, wasn't it, with you.	Malmström *et al.* 2018[43]

Supplementary Table 3 *Continued*

Metaphor label in current study	Metaphor label in original study	Example	Studies
Transformation	Inner and outer transformation	[NAME]: "I felt I was *born again*." [NAME]: "I felt like a *rebirth*."	Almegewly & Alsoraihi 2022[29]
	―― (Theme: Managing identity)	You *take on this new persona* about actually being able to talk about it as if its *second nature* and I just think it's so unhelpful.	Appleton & Flynn 2014[21]
	Transformation of Character	My affliction with breast cancer *led me to acquire a new positivity* and my life *turned into* a mission of love to spread awareness of that disease.	Abaalalaa & Ibrahim 2022[36]
Education	A learning experience	La enfermedad me enseñó a valorar la vida. [The disease *taught* me to value life.]	Magaña & Matlock 2018[31]
Job	Journey	I stopped in September, 2004. Dr. [NAME] and Dr. [NAME] both felt that I needed a chemo *vacation*.	Mijomanović 2015[35]
Burden	A heavy object	Berat nak tanggung penyakit ni, kena kuat dan banyak sabar. [It's *heavy* to *bear* this illness, I have to be strong and be more patience[t].]	Mohd Jamil *et al.* 2019[39]
	Burden	To have a relapse during ongoing treatment is *heavy*.	Hommerberg *et al.* 2020[40]
Other	Container	Create a safe atmosphere (non-judgmental, non-critical), for us to be allowed and encouraged to *vent*, rage and share the wide range of feelings we have, some of which may be seen as childish, fearful or irrational.	Mijomanović 2015[35]
