# Supplementary material for: Cancer experience in metaphors: patients, carers, professionals, students – a scoping review
Source: BMJ Support Palliat Care. 2024 May 13;14(e3):e004927. doi: 10.1136/spcare-2024-004927 (PMC11671970; doi:10.1136/spcare-2024-004927)
Supplement: online supplemental file 5 [file spcare-14-e3-s005.pdf]

Supplementary Table 4 Metaphors for treatment in selected papers

Treatment-related topics	Metaphor label in current study	Metaphor label in original study	Example	Studies
Treatment	Journey	Journey	Estoy orgullosa de todos los pasos que he dado. [I am proud of all the *steps* that I have taken.]	Gustafsson *et al.* 2020[19] Magaña & Matlock 2018[31]
		Journey	'*leaving behind*', '*entering the next phase*' or *'being on the way back*'	Gustafsson *et al.* 2020[19]
	Poison	Poison train	Then I had a break the following week and then it was time to get back on the *poison train* again and I continued like that for half a year.	Gustafsson *et al.* 2020[19]
	Violence	Violence (against the patient)	El nuevo tratamiento fue más agresivo ... [The new treatment was more *aggressive* ...]	Semino *et al*. 2017[4] Magaña & Matlock 2018[31]
		Violence (against the disease)	Physical change was not easy to assimilate, but we must appreciate that the loss of hair and other effects are minor harms if we take into account that the treatment will serve to *defeat* this disease.	Magaña 2020[30]
		Violence (against the disease)	... el tratamiento había ganado al cancer. [... the treatment had *beaten* cancer.]	Magaña & Matlock 2018[31]
		WAR (Difficulty of Treatment Metaphors)	Cancer does not kill. Treatment, however, sometimes *kills*	Abaalalaa & Ibrahim 2022[36]

Supplementary Table 4 *Continued*

Treatment-related topics	Metaphor label in current study	Metaphor label in original study	Example	Studies
Chemotherapy	Violence	Collapsing in battle	Chemo *came at* me in a fury, *like a 500 pound Sumo wrestler* determined on giving me the *beat-down* of my life. [. . .] After seven chemos, the air is going out of me, my skin is wrinkling . . .	Guité-Verret & Vachon 2021[9]
		War	In your body, the chemotherapy *fights* the cancer and not you.	Chircop & Scerri 2018[41]
		Violence	Chemotherapy is described as giving the patient's body 'a *hammering*' or 'a *battering*'.	Semino *et al.* 2017[4]
	Wave	Violence	Deep breath, before the big *wave hits* you! [chemotherapy]	Demmen *et al.* 2015[34]
	Journey	Journey	Going through chemotherapy is *like* "*walking down a long road*" (NAME) without knowing in certainty whether they will recover or not from cancer, on completing their treatment.	Chircop & Scerri 2018[41]
		Journey	Most people who have coped with and *gone through* cancer and chemotherapy say that it's the worst thing they have ever experienced.	Hommerberg *et al.* 2020[40]
		Journey	...the chemotherapy is a long arduous *journey*...	Mijomanović 2015[35]
	Personification	Militaristic metaphor	Participant (16), who is a health practitioner, said: "Chemotherapy does not treat; it only *calms down* the disease."	Albarghouthi & Klempe 2019[38]

Supplementary Table 4 *Continued*

Treatment-related topics	Metaphor label in current study	Metaphor label in original study	Example	Studies
Chemotherapy	Poison	War	Chemotherapy is *poison*… [it] destroys other organs or tears down other good cells in order to affect the bad cells.	Bodd *et al.* 2023[28]
Radiation/radiotherapy	Violence	Violence	They *hit* me with radiation for 10 days.	Demmen *et al.* 2015[34]
		Violence	what did I think all my normal little cells were doing after being *hit* by a *sledgehammer* of both toxic chemicals and radiation.	Semino *et al.* 2018[7]
		Collapsing in battle	Radiation therapy on my lungs saved my life. I don't look down on it. But finally, if it could have come by my house without *vandalizing* everything, I would have been grateful. [. . .] I'm cured. Yippee. However my insides are *burnt, shriveled, diminished, ravaged* for life.	Guité-Verret & Vachon 2021[9]
	Fire	Combinations of WAR/JOURNEY metaphors with notions of support and difficulty of treatment	The final treatment plan, radiotherapy, has started. ...and my body was *set on fire.*	Abaalalaa & Ibrahim 2022[36]
Surgery	Gardening	Other	I believe surgery was my heavenly father's way of *pruning* me so I could *blossom* and *grow* in my spiritual life. And that has been my greatest gift from cancer.	Magaña 2020[30]

Supplementary Table 4 *Continued*

Treatment-related topics	Metaphor label in current study	Metaphor label in original study	Example	Studies
Treatment decision-making	Vote	Vote	And I mean I can't possibly read up on every clinical trial and every ...everything to be as expert as them. So at some point for my own peace of mind I've got to go ok... "my *vote's* 48 percent but yours is 52."	Sinding 2014[10]
	Journey	Walk me there	So he did, but you know at that point you're saying: yea but, like can you just like give me a phone number or *walk me* [there]?...	Sinding 2014[10]
Consequence of treatment	Gift	Gift	I believe surgery was my heavenly father's way of pruning me so I could blossom and grow in my spiritual life. And that has been my greatest *gift* from cancer.	Magaña 2020[30]
Side effects of treatment--Short hair	Crown	Crown	The cruelest stage of chemotherapy is the loss of hair, which is considered the woman's *crown*. …	Abaalalaa & Ibrahim 2022[36]
