# Supplementary material for: Cancer experience in metaphors: patients, carers, professionals, students – a scoping review
Source: BMJ Support Palliat Care. 2024 May 13;14(e3):e004927. doi: 10.1136/spcare-2024-004927 (PMC11671970; doi:10.1136/spcare-2024-004927)
Supplement: online supplemental file 6 [file spcare-14-e3-s006.pdf]

Supplementary Table 5 Metaphors for people and relationships in selected papers

Aspects of Cancer	Metaphor label in current study	Metaphor label in original study	Example	Studies
The patient	Violence	Battle (Collectively affirming)	[NAME]: ... One last question, for now at least, can I officially call myself a cancer *survivor* now instead of a cancer patient?	Torres & DeBerry-Spence 2019[44]
		Violence	It is invaluable, and now they see me as a *warrior*, a *fighter*.	Demmen *et al.* 2015[34] Magaña 2020[30] Semino *et al.* 2018[7]
		War/fight	I know that now I am on a journey where I am a *conqueror* of cancer.	Bodd *et al.* 2023[28]
		War/fight	In our celebration of the *survivors*, it usually happens that we reduce their experiences with the disease to its final result, which is recovery.	Abaalalaa & Ibrahim 2022[36]
		War/fight	*Warrior heroes* who have vanquished cancer with will power and determination.	Abaalalaa & Ibrahim 2022[36] Magaña 2020[30]
	Object	Tough as old boots	'Honestly, I am *as tough as old boots*'.	Lanceley & Clark 2013[45]
		Façade metaphor	I just felt like, a *cardboard cut-out*, I felt that there was *no substance* to me anymore.	Montali *et al.* 2023[20]
	Zombie	Zombie	I feel like a *zombie* or whatever, you know slipping into a sleep and then wake up and then fall asleep again. And when you are in pain and when you don't sleep at night ... , then there isn't much dignity left".	Laursen *et al.* 2019[46]

Supplementary Table 5 *Continued*

Aspects of Cancer	Metaphor label in current study	Metaphor label in original study	Example	Studies
The patient	Intertextual metaphor--Dual Personality (Dr. Jekyll and Mr. Hyde)	Façade metaphor	The anger was very present in the first long phase of the disease... in the first three years, anger and a lot of fatigue, but I always tried to be a bit *Dr. Jekyll and Mr. Hyde*.... everything was fine outside while inside I was a volcano in other words.	Montali *et al.* 2023[20]
	Volcano	Façade metaphor	The anger was very present in the first long phase of the disease... in the first three years, anger and a lot of fatigue, but I always tried to be a bit Dr. Jekyll and Mr. Hyde.... everything was fine outside while inside I was a *volcano* in other words.	Montali *et al.* 2023[20]
Health professionals	Violence	Violence	You are now the *general* and you see your *troops killed* in *battle*.	Demmen *et al.* 2015[34]

Supplementary Table 5 *Continued*

Aspects of Cancer	Metaphor label in current study	Metaphor label in original study	Example	Studies
Social relationships	Violence	—— (Theme: Lack of comprehension and communication)	Unfortunately, some people instead of understanding what I was going through were driven by personal selfishness and *hurt* me, *hurt* me so much. My husband also *hurt* me in the period before the surgery because he didn't believe that I was sick, he thought that since I had just given birth, I was a woman in search of attention since I was at home with a small baby who cried at night, colic, and so on.	Montali *et al.* 2023[20]
	Sports	—— (Theme: Lack of comprehension and communication)	She would *bowl in* and she would be busy doing things. She wouldn't sit and just talk or just wouldn't sit and have a cup of tea ... but I think, she wasn't doing it intentionally, I know that. It was just her response to a situation that she felt had just spun completely out of control.	Montali *et al.* 2023[20]
		—— (Theme: Social relationships as empowerment resources)	It was a group of people who were a *team*, and this is very important; this friend of mine came out of it [cancer] even though she had a terrible experience.	Montali *et al.* 2023[20]

Supplementary Table 5 *Continued*

Aspects of Cancer	Metaphor label in current study	Metaphor label in original study	Example	Studies
Social relationships	Distance	—— (Theme: Isolation, marginalisation and self-isolation)	I have a couple of people that were very shocked by the diagnosis, probably not as much as me but quite shocked anyway. yeah, a couple of them have taken a while to connect back with, as such, they have *drifted away*, it's all a bit much, it was all a bit hard to deal with.	Montali *et al.* 2023[20]
		—— (Theme: Isolation, marginalisation and self-isolation)	There are those who instead *came out* of my life or in any case put themselves *in a second position*, who *took*, let's say, *a lateral position* in my life.	Montali *et al.* 2023[20]
		Proximity	I was lucky ... and supported especially by the people *close* to me who gave me a lot of help and I ... I'm modest ... I was able with my character to react quite well".	Montali *et al.* 2023[20]
		—— (Theme: Isolation, marginalisation and self-isolation)	you don't want everyone to see you at your absolute lowest ... I *withdrew* into myself. Like I was, well I know that I *withdrew* from a lot of people as well because I wasn't feeling up to being social.	Montali *et al.* 2023[20]
		—— (Theme: Social relationships as empowerment resources)	Even relations who are living, you know thousands of kilometers away, would keep in contact and phone weekly, you know? An influx, an influx of fun emails and things like that you know? That really skyrocketed ... it really did *pull*, *pull* everybody together a lot.	Montali *et al.* 2023[20]

Supplementary Table 5 *Continued*

Aspects of Cancer	Metaphor label in current study	Metaphor label in original study	Example	Studies
Patient-patient relationship	Violence	Violence	share nicely our *Camp Commandants*. . .. and I bought it at a shop, cos you just don't listen do you *Colonel*...	Semino *et al.* 2018[7]
		Violence	'*Captain*' *for one another*; one particular patient says that she would *'promote' another* if he had not already 'reached *top rank*'.	Semino *et al.* 2017[4]<br>Semino *et al.* 2018[7]
Heath professional-Patient relationship	Violence	Violence	A patient describes a successful outcome in a consultation as '*winning* that *battle*', while another uses the expression 'twin *attack*' to refer to how two family members managed to obtain a medical appointment.	Semino *et al.* 2017[4]
		Violence	I now have another thing to *beat* my surgeon *up* about.	Semino *et al.* 2018[7]
		Violence/Protect	we (doctors) must focus on the *protection* of our patients and on advancing palliative care.	Demmen *et al.* 2015[34]
Carer-healthcare system relationship	Violence	Violence	It seems to me that while my husband fights cancer, I am *fighting* the system.	Demmen *et al.* 2015[34]
Heath professional-higher authority relationship	Violence	Violence	*fighting* with health authorities and PCTs for [...] funding.	Demmen *et al.* 2015[34]
